# Neonatal outcomes following previable rupture of membranes below 23 weeks’ gestation

**DOI:** 10.1007/s00431-025-06324-0

**Published:** 2025-07-25

**Authors:** Agnes Grill, Fanny Mikula, Sophie Jansen, Lisa Klein, Judith Rittenschober-Boehm, Harald Leitich, Alex Farr, Angelika Berger, Katharina Goeral

**Affiliations:** 1 Division of Neonatology, Intensive Care and Neuropediatrics, Department of Pediatrics and Adolescent Medicine, Comprehensive Center for Pediatrics, Medical University of Vienna, Vienna, Austria; 2 Division of Obstetrics and Fetomaternal Medicine, Department of Obstetrics and Gynecology, Comprehensive Center for Pediatrics, Medical University of Vienna, Vienna, Austria; 3 Department of Neonatology, Women’s and Children’s Clinic (Med Campus IV), Kepler University Hospital, Linz, Austria

**Keywords:** Preterm premature rupture of membranes, Neonate, Extreme prematurity, Morbidity, Survival

## Abstract

This study aimed to provide institution-specific, up-to-date data to improve counseling for pregnant women experiencing preterm premature rupture of membranes (pPROM) before or at the limit of viability. We conducted a retrospective cohort study (2009–2022) of preterm infants with pPROM < 23 + 0 weeks’ gestation who received active neonatal care. The primary outcomes were survival and survival without severe short-term morbidity (defined as IVH III, PVHI, cPVL, and/or ROP). Data from 109 patients with a median gestational age at pPROM of 21.6 weeks and a median gestational age at delivery of 25.4 weeks were analyzed. The overall survival rate was 69.7%, and survival without short-term severe morbidity was 51.4%. Both outcomes improved significantly with increasing gestational age at birth. Early-onset neonatal sepsis (culture-positive and culture-negative) occurred in 18.3% of cases, with its risk inversely correlated with both gestational age at birth and latency until delivery. Higher gestational age at pPROM (aOR 1.945 (95% CI 1.193–3.171) per week) and longer latency until delivery (aOR 1.115 (95% CI 1.060–1.173) per day) were significant predictors of survival without severe morbidity. *Conclusion*: Our study provides valuable, institution-specific data on outcomes for infants born after pPROM < 23 + 0 weeks’ gestation. Gestational age at pPROM and latency until delivery are key predictors of survival and survival without severe morbidity in preterm infants. These results offer important insights for counseling pregnant women experiencing pPROM at or near the limit of viability.

**Table:** 

**What is Known:** • *Counseling patients after previable pPROM 23+0 weeks is challenging due to limited data.* • *Outcomes vary widely between centers.*
**What is New:** • *Institution-specific survival after previable pPROM with active management was 69.7%; gestational age at pPROM and latency until delivery independently predicted intact survival.* • *Early-onset neonatal sepsis risk decreased with longer latency despite prolonged membrane rupture.*

**Supplementary Information:**

The online version contains supplementary material available at 10.1007/s00431-025-06324-0.

## Introduction

Premature preterm rupture of membranes (pPROM) before or at the limit of viability complicates 0.1–0.7% of pregnancies and is associated with significant neonatal mortality and morbidity [1–4]. The management of pPROM at this stage of pregnancy differs markedly from later cases. At this early stage, the risks of a wait-and-see approach must be carefully weighed against the decision to terminate the pregnancy. The threshold of viability, which corresponds to approximately 22 + 0 weeks’ gestation, represents the stage at which fetal maturity offers a limited chance of survival outside the womb. Complications for the mother and fetus are severe and include chorioamnionitis, fetal loss, pulmonary hypoplasia, deformities due to restriction, and issues related to extreme prematurity in surviving infants [1, 4].

The time interval between membrane rupture and delivery is a critical factor in determining outcome in previable pPROM. Approximately 50% of these pregnancies result in delivery shortly after pPROM [5–7]. By 28 days post-rupture, the vast majority of pregnancies have delivered [4, 8]. The duration of latency between pPROM until delivery is inversely related to gestational age at admission [4, 5, 9]. However, in some cases, the fetal membranes spontaneously reseal; in others, amniotic fluid loss persists but is partially replenished.

At the threshold of viability, when active care becomes an option, the neonatologist plays a critical role in providing realistic guidance on the unborn child’s mortality and morbidity risks, considering both the timing of rupture and latency until delivery, to help parents make informed decisions. Counseling patients with pPROM at this stage is challenging due to limited data, mostly from small, single-center experiences, varying gestational ages, and the retrospective nature of many studies conducted before the introduction of modern therapies [1]. Further difficulties arise from differences in in- and exclusion criteria, diagnostic standards, and limited adjustment of confounding factors [1, 10, 11], resulting in highly variable outcomes across studies, with neonatal survival rates ranging from 23 to 95% [6, 7, 11–18], and survival without severe morbidity from 26 to 81% [7, 11, 16, 18, 19]. Study design differences furthermore hinder the comparability of outcomes and their applicability to individual cases.

Ongoing research into maternal and neonatal outcomes is essential as gestational viability continues to decrease with advances in obstetrics and neonatology. We believe that reliable guidance and decision-making can only be based on a center’s own data [17, 20].

This study therefore aimed to generate institution-specific, up-to-date data to improve counseling for pregnant women experiencing previable pPROM.

## Materials and methods

### Study setting

The study was conducted at our tertiary perinatal center, which has 54 neonatal care beds and manages approximately 2.500–2.800 deliveries annually [21]. Each year, the center cares for around 90 infants < 1000 g and 180 infants < 1500 g, with survival rates of around 82% and 90%, respectively.

For this analysis, we included cases of pPROM before 23 + 0 weeks that received active neonatal care. In twin pregnancies, only the fetus affected by pPROM was included. The study period spanned from June 1, 2009, to December 31, 2022. Cases with chromosomal abnormalities and major malformations were excluded.

The study protocol was approved by the Institutional Review Board (1415/2014). Patient consent was not required due to the retrospective nature of the study.

### Obstetrical standard of care

The diagnosis of pPROM was confirmed by visualizing amniotic fluid or detecting insulin-like growth factor binding protein-1 or placental alpha microglobulin-1 in vaginal fluid. Our management protocol included antenatal corticosteroids (betamethasone 12 mg, two doses 24 h apart) and a 7-day antibiotic regimen (ampicillin 4 g, three times daily). Starting in 2017, a single 1-g dose of azithromycin was added. Labor tocolysis with Atosiban was given for at least 48 h. Since 2015, magnesium sulfate was used for fetal neuroprotection during the last 24 h before delivery. Oligohydramnios was defined as an amniotic fluid index < 5 cm, and anhydramnios as a deepest pocket < 1 cm after pPROM. Chorioamnionitis was suspected in case of maternal fever > 38 °C, tachycardia > 100/min, purulent vaginal discharge, uterine tenderness, or fetal tachycardia > 160/min. In cases of C-section, placental and membrane cultures were taken to detect bacterial or fungal pathogens.

As part of our routine perinatal care, our center follows a proactive approach with planned caesarean delivery during routine service hours whenever possible. For patients with pPROM below 23 weeks, if spontaneous labor has not occurred, delivery is generally scheduled after a latency period of 3–4 weeks, following parental counseling and evaluation of key factors such as amniotic fluid volume and signs of infection. For extremely premature infants, caesarean delivery is the preferred mode of delivery at our center.

### Neonatal standard of care

In the delivery room, standard management included early high-flow continuous positive airway pressure (CPAP) using a Benveniste valve. Prophylactic surfactant was administered via a thin catheter using the less invasive surfactant administration (LISA) technique for spontaneously breathing infants born at ≤ 27 + 6 weeks. Alternatively, invasive surfactant was given if intubation was required. Early rescue surfactant was provided for infants born ≤ 29 + 6 weeks when indicated.

For all infants born at ≤ 27 + 6 weeks, blood cultures were obtained, and prophylactic broad-spectrum antibiotics (ampicillin and gentamicin) started for at least 48 h.

### Descriptive data and short-term outcome

Obstetric and perinatal variables were extracted from patient records. Neonatal pH levels from blood gas analyses in the first hour after birth were analyzed. Data on respiratory outcomes included surfactant use, duration of mechanical ventilation, and complications such as pulmonary hypertension and pneumothorax. Bedside echocardiography was performed to screen for pulmonary hypertension in the first days of life, with treatment primarily including inhaled nitric oxide, intravenous milrinone, and in severe cases, inhaled iloprost and intravenous sildenafil.

Mortality prior to hospital discharge was analyzed. Early-onset neonatal sepsis (EONS) was defined as either culture-positive EONS characterized by a positive blood culture within 72 h or culture-negative EONS, in which infants exhibited clinical signs and elevated inflammatory markers and (e.g., CRP, IL-6) prompting continued antibiotic therapy despite negative blood cultures. This operational definition is consistent with current clinical practice and literature [22, 23]. Moreover, the following morbidities were accessed: severe cerebral morbidity was indicated by grade III intraventricular hemorrhage (IVH), periventricular hemorrhagic infarction (PVHI), or cystic periventricular leukomalacia (cPVL); bronchopulmonary dysplasia (BPD) was defined as the need for oxygen support at 36 weeks postmenstrual age; severe retinopathy of prematurity (ROP) was classified as grade 3 or higher; surgically treated persistent ductus arteriosus (PDA); necrotizing enterocolitis (NEC) above Bell’s stage 2. Finally, “severe morbidity” was defined as a composite outcome of severe cerebral morbidity (IVH III, PVHI, cPVL), and/or severe ROP.

### Statistical analysis

For statistical analysis, we used SPSS Statistics, version 27 for Mac (IBM Corporation, NY, USA). A two-sided *p*-value < 0.05 was considered statistically significant. Demographic data are presented as means ± standard deviation (SD) or median and interquartile range (IQR) for quantitative variables and as counts and percentages for categorical variables. The patient groups were stratified based on survival status and gestational age at birth. Differences between groups were compared using *t*-test, Mann–Whitney *U* test, ANOVA, or chi-square test, where appropriate.

The association between latency until delivery and EONS was investigated using logistic regression. Multivariable logistic regression analysis was also performed to identify relevant factors for counseling pregnant women, including the factors gestational age at pPROM, latency until delivery, birthweight percentile, gender, twin status, and the presence of anhydramnios, which were selected based on expert opinion and known association in the literature. To test robustness, a sensitivity analysis excluding twin pregnancies was performed. To account for changes in management protocols and advances in neonatal care over time, management era (early 2009–2015 vs. late 2016–2022) was included as a covariate. Results of regression analyses are presented as adjusted odds ratios (aOR) with corresponding 95% confidence intervals (CI).

## Results

In our retrospective analysis, we included 109 live-born preterm infants delivered after pPROM < 23 + 0 weeks’ gestation. Of these infants, 92 patients (84.4%) were from singleton pregnancies, and 17 patients (15.6%) were from twin pregnancies.

Table 1 presents maternal and neonatal characteristics of the entire cohort and by neonatal survival status. Maternal characteristics showed no significant differences between survivors and non-survivors. The median gestational age at pPROM was 21.6 weeks [IQR 20.6–22.3], median gestational age at delivery 25.4 weeks [IQR 23.9–26.4] and mean birthweight 771 ± 219 g. Gestational age at pPROM was comparable between surviving and non-surviving infants. Gestational age at birth and birthweight were significantly higher in the group of survivors, by 1.5 weeks and 189 g, respectively. The median latency between rupture of membranes and delivery was 26 days [IQR 17–40] without significant differences between the groups. Apgar values and umbilical cord pH were significantly worse in non-survivors.

**Table 1:** Maternal and neonatal characteristics by survival status

	Entire cohort *n* = 109	Survivors *n* = 76	Non-survivors *n* = 33	*p*-value
Maternal characteristics				
Maternal age (yrs) *	31.6 [28.0–36.1]	31.2 [27.8–35.4]	31.9 [28.3–37.4]	0.389
Maternal antibiotics *n* (%)	109 (100.0)	76 (100.0)	33 (100.0)	NA
Tocolytics *n* (%)	107 (98.2)	76 (100.0)	31 (93.9)	0.09
Antenatal steroids *n* (%)	109 (100.0)	76 (100.0)	33 (100.0)	NA
C-section *n* (%)	93 (85.3)	67 (88.2)	26 (78.8)	0.242
Maternal CRP mg/dL *	0.84 [0.31–2.15]	0.67 [0.31–1.93]	1.01 [0.30–3.73]	0.319
Maternal leukocytes G/L *	12.67 [10.19–15.45]	12.55 [10.27–15.22]	12.93 [10.10–17.00]	0.666
Suspected chorioamnionitis *n* (%)	25 (22.9)	16 (21.1)	9 (27.3)	0.478
Oligohydramnios *n* (%)	55 (51.4)	39 (52.0)	16 (50.0)	0.511
Anhydramnios *n* (%)	35 (32.7)	26 (34.7)	9 (28.1)	
Placental abruption *n* (%)	6 (5.5)	3 (3.9)	3 (9.1)	0.364
Cord prolapse *n* (%)	2 (1.8)	1 (1.3)	1 (3.0)	1.000
Maternal sepsis *n* (%)	6 (5.6)	3 (4.0)	3 (9.1)	0.367
Positive placental culture^#^*n*(%)	35 (45.5)	26 (46.4)	9 (42.9)	0.779
Neonatal characteristics				
GA at pPROM (weeks) *	21.6 [20.6–22.3] min–max: 12.1–22.9	21.6 [20.7–22.4] min–max: 12.1–22.9	21.4 [20.1–22.2] min–max: 16.9–22.9	0.389
GA at birth (weeks) *	25.4 [23.9–26.4] min–max: 22.6–29.6	25.8 [24.0–26.9] min–max: 22.9–29.6	24.3 [23.5–25.4] min–max: 22.6–29.1	**0.001**
Latency time pPROM to birth (days) *	26 [17–40] min–max: 0–94	29 [17–41] min–max: 2–94	19 [13–32] min–max: 0–72	0.057
0–7 days *n* (%)	8 (6.4)	5 (6.6)	2 (6.1)	0.197
8–14 days *n* (%)	15 (13.8)	9 (11.8)	6 (18.2)	
15–21 days *n* (%)	18 (36.7)	9 (11.8)	9 (27.3)	
22–28 days *n* (%)	21 (19.3)	15 (19.7)	6 (18.2)	
> 28 days *n* (%)	48 (44.0)	38 (50.0)	10 (30.3)	
Gender (male, %)	65 (59.6)	46 (60.5)	19 (57.6)	0.773
Birthweight (g) °	771 ± 219 min–max: (350–1305)	828 ± 221 min–max: (432–1305)	639 ± 146 min–max: (350–1046)	** < 0.001**
APGAR 1 min	7 [6–8]	7 [6–8]	6 [4–7]	** < 0.001**
APGAR 5 min < 7 (%)	8 [8–9]	8 [8–9]	8 [5–8]	** < 0.001**
APGAR 10 min	9 [9–9]	9 [9–9]	9 [7–9]	**0.001**
Cord blood pH *	7.34 [7.28–7.39]	7.36 [7.32–7.39]	7.28 [7.21–7.36]	** < 0.001**
Neonatal pH *	7.16 [7.07–7.23]	7.18 [7.10–7.23]	7.12 [7.00–7.22]	0.097

Abbreviations: *GA*, gestational age; *pPROM*, premature preterm rupture of membranes. Legend: °mean ± SD; *median including [IQR]; ^#^only patients delivered by C-section

Table 2 describes neonatal short-term outcome, mortality, and short-term morbidity in relation to the gestational week at birth. Surfactant was administered in 99.1%, and mechanical ventilation was necessary in 67.6% of patients in the first week of life. The rates for pneumothorax and pulmonary hypertension were 10.9% and 47.7%, respectively. Pulmonary outcomes did not differ significantly across the different gestational ages at birth.

**Table 2:** Neonatal short-term outcome, mortality, and morbidities by gestational age at birth

GA at birth	Entire cohort *n* = 109	GA 22 ^0–7 ^*n* = 2	GA 23 ^0–7^ *n* = 29	GA 24 ^0–7 ^*n* = 17	GA 25 ^0–7 ^*n* = 21	GA 26 ^0–7 ^*n* = 21	GA 27 ^0–7 ^*n* = 12	GA 28 ^0–7 ^*n* = 5	GA 29 ^0–7^ *n* = 2
Pulmonary outcome									
Surfactant received *n* (%)	107 (99.1)	2 (100.0)	28 (100.0)	17 (100.0)	21 (100.0)	21 (100.0)	12 (100.0)	4 (80.0)	2 (100.0)
Mechanical ventilation first 7 doL *n* (%)	73 (67.6)	2 (100.0)	23 (82.1)	12 (70.6)	11 (52.4)	14 (66.7)	8 (66.7)	2 (40.0)	1 (50.0)
HFOV first 7 doL *n* (%)	46 (42.6)	2 (100.0)	11 (39.3)	7 (41.2)	7 (33.3)	12 (67.1)	5 (41.7)	1 (20.0)	1 (50.0)
Pneumothorax first 7 doL *n* (%)	11 (10.9)	0 (0.0)	4 (15.4)	1 (6.7)	1 (5.0)	3 (14.3)	2 (16.7)	0 (0.0)	0 (0.0)
Pulmonary hypertension (%)	52 (47.7)	0 (0.0)	14 (48.3)	9 (52.9)	9 (42.9)	8 (38.1)	9 (75)	2 (40.0)	1 (50.0)
Neonatal morbidities									
EONS any *n* (%)	19 (18.3)	1 (50.0)	9 (36.0)	3 (18.8)	4 (19.0)	1 (4.8)	1 (8.3)	0 (0.0)	0 (0.0)
Culture-positive	5 (4.8)	0 (0.0)	1 (4.0)	2 (12.5)	1 (4.8)	1 (4.8)	0 (0.0)	0 (0.0)	0 (0.0)
Culture-negative	14 (13.5)	1 (50.0)	8 (32.0)	1 (6.3)	3 (14.3)	0 (0.0)	1 (8.3)	0 (0.0)	0 (0.0)
PDA with surgical intervention *n* (%)	6 (6.7)	0 (0.0)	3 (14.3)	1 (7.7)	0 (0.0)	1 (4.8)	1 (10.0)	0 (0.0)	0 (0.0)
NEC with surgical intervention *n* (%)	11 (11.5)	0 (0.0)	5 (21.7)	2 (14.3)	3 (15.0)	1 (4.8)	0 (0.0)	0 (0.0)	0 (0.0)
IVH III/PVHI *n* (%)	13 (13.5)	0 (0.0)	3 (13.0)	3 (21.4)	4 (20.0)	1 (4.8)	2 (18.2)	0 (0.0)	0 (0.0)
cPVL *n* (%)	0 (0.0)	0 (0.0)	0 (0.0)	0 (0.0)	0 (0.0)	0 (0.0)	0 (0.0)	0 (0.0)	0 (0.0)
BPD 36 GA *n* (%)	22 (29.3)	0 (0.0)	7 (43.8)	3 (37.5)	2 (14.3)	7 (35.0)	2 (20.0)	0 (0.0)	1 (100.0)
ROP with intervention *n* (%)	15 (18.5)	0 (0.0)	9 (52.9)	3 (25.0)	2 (14.3)	1 (4.8)	0 (0.0)	0 (0.0)	0 (0.0)
Outcome									
Survival *n* (%)	76 (69.7)	1 (50.0)	15 (51.7)	9 (52.9)	14 (66.7)	21 (100.0)	10 (83.3)	5 (100.0)	1 (50.0)
Survival without severe morbidity *n* (%)	56 (51.4)	1 (50.0)	6 (20.7)	4 (23.5)	11 (52.4)	20 (95.2)	8 (66.7)	5 (100.0)	1 (50.0)
Discharge doL *	99 [85–114]	89 [89–89]	129 [114–139]	108 [100–119]	93 [88–98]	93 [78–111]	75 [66–82]	70 [66–]	NA
Discharge GA (weeks) *	39.0 [38.14–41.29]	35.57 [35.57–35.57]	42.29 [39.36–43.14]	39.43 [38.75–41.64]	38.72 [38.04–39.25]	39.50 [37.54–42.25]	38.50 [37.0–39.04]	38.57 [37.71–]	NA

*doL* day of life, *EONS* early-onset neonatal sepsis (culture-positive and culture-negative), *GA* gestational age (in weeks + days), *LISA* less invasive surfactant administration, *pPROM* preterm premature rupture of membranes, *PVHI* periventricular hemorrhagic infarction. Legend: *median including [IQR]; severe morbidity: IVH III, PVHI, cPVL, BPD, and/or ROP

**Fig. 1:**
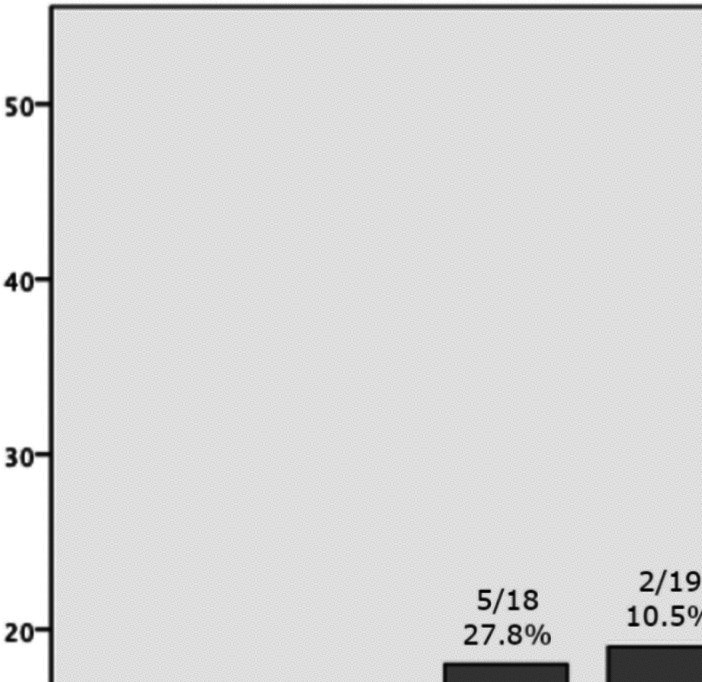
The incidence of EONS relative to the number of days of latency between pPROM and birth. Abbreviations: EONS, early-onset neonatal sepsis (culture-positive and culture-negative)

The overall rate of EONS in our study group was 18.3%, comprising 4.8% culture-positive and 13.5% culture-negative cases. The risk of EONS—particularly culture-negative EONS—significantly decreased with increasing gestational age at birth (*p* = 0.002 and 0.003, respectively). Figure 1 illustrates the rates of EONS stratified by week of latency until delivery. Logistic regression analysis revealed an OR for latency of 0.967 (95% CI 0.935–1.000), indicating that each additional day of latency until delivery was associated with a 3.3% reduction in the odds of EONS.

The most frequent short-term morbidities were BPD, ROP, and IVH III/PVHI with 29.3%, 18.5%, and 13.5%, respectively. The overall survival rate was 69.7%, while overall survival without severe morbidity was 51.4%. Consequently, 73.7% of survivors did not experience severe morbidity. Both survival and survival without severe morbidity increased significantly with increasing GA at birth. Table 3, along with Supplemental Tables A and B, presents the raw data on survival and survival without severe morbidity, stratified by gestational age at pPROM and at birth.

**Table 3:** Survival and survival without severe morbidity in relation to GA at pPROM and GA at birth

GA at pPROM	Latency (days)	GA at birth	Survival	Survival without severe morbidity
GA 12 ^0–7^ *n* = 1	94 (94–94)	25.57 (25.57–25.57)	1/1 (100.0%)	1/1 (100%)
GA 15 ^0–7 ^*n* = 2	76 (75–)	26.72 (26.57–)	2/2 (100.0%)	2/2 (100.0%)
GA 16 ^0–7^ *n* = 2	62 (51–)	25.57 (24.00–)	1/2 (50.0%)	0/2 (0.0%)
GA 17 ^0–7^ *n* = 4	50 (40–69)	24.57 (23.33–27.32)	1/4 (25.0%)	1/4 (25.0%)
GA 18 ^0–7^ *n* = 4	58 (57–61)	26.29 (26.18–27.36)	4/4 (100.0%)	4/4 (100.0%)
GA 19 ^0–7^ *n* = 2	41 (31–)	25.50 (24.29–)	1/2 (50.0%)	1/2 (50.0%)
GA 20 ^0–7^ *n* = 17	38 (25–50)	26.00 (23.72–27.43)	10/17 (58.8%)	8/17 (47.1%)
GA 21 ^0–7^ *n* = 31	26 (16–34)	25.14 (23.57–26.43)	24/31 (77.4%)	16/31 (51.6%)
GA 22 ^0–7^ *n* = 46	19 (9–25)	24.86 (23.79–25.90)	32/46 (69.6%)	23/46 (50.0%)
**Total *n* = 109**	**26 (17–40)**	**25.43 (23.86–26.43)**	**76/109 (69.7%)**	**56/109 (51.4%)**

*GA* gestational age (in weeks + days), *pPROM* preterm premature rupture of membranes. Legend: severe morbidity: IVH III, PVHI, cPVL, and/or ROP

In the context of prenatal counseling, multivariable logistic regression analysis identified two significant predictors for both survival and survival without severe morbidity (Table 4): higher GA at the time of pPROM and a longer latency until delivery. The aOR for GA at pPROM was 1.945 (95% CI 1.193–3.171), indicating that with each additional week of GA at pPROM, the odds of survival without severe morbidity nearly doubled. Longer latency between pPROM and birth was associated with a further 11% improvement in these odds per day (aOR 1.115; 95% CI 1.060–1.173). Weight percentile was also an independent predictor (aOR 1.035; 95% CI 1.012–1.058). Twin status, gender, and anhydramnios were not identified as predictors. A sensitivity analysis excluding twins yielded essentially unchanged results, confirming the robustness of our model (data not shown). Management era was significantly associated with primary outcomes: for survival, aOR 0.384 (95% CI 0.148–0.998; *p* = 0.050), and for survival without severe morbidity, aOR 0.155 (95% CI 0.053–0.459; *p* = 0.001), indicating 62% and 85% lower odds of adverse outcomes in the late period, respectively.

**Table 4:** Results of multivariable logistic regression analysis

		Survival			Survival without severe morbidity		
		Sign	aOR	95% CI	Sign	aOR	95% CI
Factor	GA at pPROM	**.049**	1.592	1.002–2.538	**.008**	1.945	1.193–3.171
	Latency until delivery	**.016**	1.059	1.011–1.109	**.000**	1.115	1.060–1.173
	Weight percentile	**.026**	1.023	1.003–1.043	**.003**	1.035	1.012–1.058
	Twins	.980	.983	.244–3.963	.452	1.759	.404–7.668
	Gender	.914	1.056	.397–2.810	.119	.419	.140–1.252
	Anhydramnios	.113	.354	.098–1.278	.569	.659	.156–2.775
	Management era	**.050**	.384	.148–.998	**.001**	.155	.053–.459

*GA* gestational age, *aOR* adjusted odds ratio, *pPROM* preterm premature rupture of membranes. Legend: model adjusts for all variables listed; sensitivity analysis excluding twins yielded similar estimates; management era split into early (2009–2015) and late years (2016–2026); severe morbidity: IVH III, PVHI, cPVL, and/or ROP

## Discussion

The aim of our study was to evaluate current, institution-specific neonatal outcomes after previable pPROM at a large tertiary perinatal center, based on a proactive management strategy tailored to pPROM. Our primary findings include a survival rate of 69.7% and a survival without severe morbidity of 51.4% in a cohort with a median gestational age at birth of 25.4 weeks and pPROM latency until delivery of nearly 4 weeks. Importantly, the purpose of this study was not to advocate for a particular delivery timing strategy, but rather to identify key prognostic factors that may inform prenatal counseling.

Counseling women with pPROM before or at the limit of viability is particularly challenging due to the wide variability in reported neonatal survival rates (ranging from 20 to 95%) and the equally high variability in the incidence of severe morbidity among survivors (30–100%) [12–15]. These substantial differences underscore the importance of using institution-specific data to guide counseling and decision-making.

In our study, we evaluated 109 cases with pPROM < 23 + 0 and found no distinct difference in outcomes between pPROM before and after 22 weeks (Table 3). While some reports indicate survival differences as high as 43.3% (14.4 versus 57.7%) using this 22-week threshold [1], others, such as Kiver et al., found no significant variation based on the timing of membrane rupture. [11] Despite the absence of a clear cutoff, gestational age at pPROM remains a key prognostic factor for perinatal outcomes in our study cohort, shown by logistic regression analysis which is consistent with the existing literature [7, 17, 19].

Studies with the highest survival rates up to 90% typically report longer latency until delivery and, consequently, higher gestational ages at birth [11, 15, 16]. Herzlich et al. found a 79% survival rate after pPROM at 17–23 weeks, with a median latency of 49 days, and a prevalence of severe morbidities (IVH III, PVHI, ROP, hearing impairment) among survivors of only 31.6%. [15] In our cohort, the median latency was 26 days, which—due to our local management strategy—was shorter than the 38–49 days reported in other studies [11, 15, 16]. Among survivors, 26.3% experienced severe morbidities, including IVH III, PVHI, cPVL, and/or ROP.

There is general concern that EONS may occur following prolonged rupture of membranes. Comparing our results with the studies mentioned above is challenging due to methodological differences. Reported culture-positive EONS rates vary from 5% [19] to 16.7% [15]. Importantly, both studies excluded patients with latency less than 7 days, a subgroup in which our study observed the highest EONS rate (Fig. 1). In contrast, Drassinower reported an overall neonatal sepsis rate of 15.5% (including both culture-positive and -negative cases throughout the entire hospital stay) [24], while Kiver observed an EONS infection rate as high as 56.8%—albeit without a clear definition—without a statistically significant impact on intact survival [11]. Previous studies investigating latency after pPROM as an independent risk factor for neonatal sepsis found no clear evidence of an association [6, 24]. Despite the assumption of infection in neonates following pPROM, multiple studies observed no difference in EONS incidence between latency until delivery of < 24 h and > 7 days [6, 19, 24]. In our study cohort, we did not observe an increase in the rate of EONS with increasing latency; in fact, the odds of EONS decreased with longer latency until delivery. This correlation suggests that neonates who develop infections are more likely to be delivered shortly after pPROM, while those without infection tend to remain in utero for a longer duration.

This finding, while counterintuitive, challenges the long-standing assumption that prolonged latency inherently increases infection risk. It suggests that, in the absence of clinical signs of infection, continued expectant management may be associated with lower odds of EONS. This underscores the need for further prospective studies to better delineate which patients may benefit from extended latency.

It should be noted that our cohort was managed proactively, with elective caesarean delivery typically scheduled 3–4 weeks after pPROM, which may limit the generalizability of our findings to patient populations with different clinical strategies.

In our cohort, 22.9% of mothers with pPROM developed clinical chorioamnionitis, a condition that can lead to neonatal sepsis and significantly impact both mortality and morbidity [25]. We found no difference in the incidence of clinical chorioamnionitis between survivors and non-survivors. Furthermore, neither the maternal biomarkers nor the incidence of positive placental cultures differed between survivors and non-survivors. Recent findings from our group demonstrated the limited predictive value of maternal CRP and leukocyte levels for EONS following early pPROM [26].

Our study found no statistically significant differences in pulmonary outcomes across gestational ages, suggesting that increased gestational age neither reduces pulmonary morbidity nor increases pulmonary hypertension, even with prolonged latency after pPROM. Conflicting reports exist on latency and pulmonary hypoplasia. For example, a Korean Neonatal Network analysis of 884 cases found that latency ≥ 7 days did not affect survival but did raise early pulmonary hypertension and BPD risk [6]. In our cohort, pulmonary hypertension was observed in 47.7% of cases, higher than the 13.8–38% reported elsewhere [6, 13, 15, 19]. This elevated rate may reflect our focus on very early gestational ages and the use of a rigorous, protocolized bedside echocardiography screening approach, including asymptomatic infants. This systematic screening may have identified milder or transient forms of pulmonary hypertension that are often missed in studies relying on clinically triggered assessments. While this approach increases detection sensitivity, it may reduce comparability with studies employing different diagnostic thresholds or selective screening strategies. These factors should be considered when comparing across cohorts. Prolonged latency may simply reflect earlier pPROM rather than being an independent risk factor [15].

Lung maturity in the second trimester largely depends on the amount of amniotic fluid available. While limited, existing literature often identifies oligohydramnios as a risk factor for mortality, early pulmonary hypertension, BPD, and chorioamnionitis [6, 8, 14]. A recent study concluded that oligohydramnios, rather than pPROM itself, is the key risk factor for poor outcomes, with higher odds of pulmonary hypertension, ROP, and neonatal death [27]. A review by Waters and Mercer reported that persistent oligohydramnios had a sensitivity of 52–100%, specificity of 41–82%, and a negative predictive value of 89–100% for pulmonary hypoplasia [1]. However, this was not consistently linked to poor outcomes [13, 18]. In our study, the rate of oligohydramnios was 84.1%, considerably higher than the 20.4–60% reported in the literature [6, 8, 18, 27]. Reported definitions vary, from < 2 cm [8, 18] to < 5 cm [6, 27], and it is unclear how frequently amniotic fluid volume was assessed [11]. We measured the amount of amniotic fluid at three time points—at rupture/admission, during the course of pregnancy, and shortly before birth—using the lowest recorded value for our analysis. Fluid levels fluctuated considerably between timepoints, raising concerns that we were capturing only isolated snapshots [11]. Persistent severe anhydramnios [13] was rare, and oligohydramnios was not a significant prognostic factor in our analysis.

Fetal MRI to assess lung volume may offer a more reliable prognostic alternative to amniotic fluid measurements [28]; however, further investigation and validation in large pPROM cohorts are needed.

### Strengths and limitations

Key limitations of our study include its retrospective design and relatively small sample size. Nevertheless, our cohort of 109 patients with pPROM < 23 + 0 weeks is comparable to those reported in other studies. A major strength is the clearly defined, homogeneous group, which enhances the value of our findings for parental counseling at the threshold of viability. We acknowledge that the exclusion of pregnancies without active neonatal care introduces a potential selection bias and may lead to underestimation of mortality at the earliest gestational ages. Furthermore, as our study is based solely on a NICU database including only infants admitted for active care, we lacked access to data on the total number of pregnancies with early pPROM managed at our institution. This absence of data on cases that miscarried, opted for comfort care, or were stillborn limits the representativeness of our cohort.

Our institutional protocol—elective delivery 3–4 weeks after pPROM < 23 weeks—is not aligned with most international guidelines (e.g., ACOG, RCOG, CNGOF), which recommend continued expectant management through at least 34 weeks [4, 20]. Importantly, our data show no benefit from early induction; rather, longer latency correlated with improved outcomes, suggesting that expectant management may be more beneficial in the absence of clinical contraindications. This discrepancy highlights the need to critically reevaluate our local protocols and align them more closely with international best practices.

A further limitation is that our study is based solely on a NICU database including infants who remained in utero after early pPROM and received active care after reaching viability. Thus, the broader obstetric cohort—including stillbirths and cases managed with compassionate care—is not represented. This may lead to selection bias and limits the generalizability of our findings regarding overall perinatal outcomes.

Although management era was a significant predictor in our models, adjusting for it did not alter the primary conclusions regarding our main exposure and outcome associations. Furthermore, our database included only births before 30 + 0 weeks; given the few patients at 28–29 weeks, it is unlikely that many cases were omitted.

## Conclusion

Our study provides valuable institution-specific data on outcomes for infants born after pPROM before 23 + 0 weeks’ gestation. It highlights gestational age at pPROM and latency until delivery as key predictors of survival and survival without severe morbidity in preterm infants. The overall survival rate was 69.7%, with 73.7% of survivors free from severe morbidity. Our results offer critical insights for counseling pregnant women facing pPROM at or near the limit of viability.

## Supplementary Information

Below is the link to the electronic supplementary material.

Supplementary Material 1 (DOCX 17.1 KB)

## Data Availability

Data are provided within the manuscript and supplementary files. Additional data are available from the corresponding author upon reasonable request.

## Abbreviations

ANOVA: Analysis of variance
BPD: Bronchopulmonary dysplasia
CI: Confidence interval
CPAP: Continuous positive airway pressure
CRP: C-reactive protein
cPVL: Cystic periventricular leukomalacia
C-section: Caesarean section
EONS: Early-onset neonatal sepsis
GA: Gestational age
IQR: Interquartile range
IVH: Intraventricular hemorrhage
LISA: Less invasive surfactant administration
NEC: Necrotizing enterocolitis
aOR: Adjusted odds ratio
PDA: Persistent ductus arteriosus
pPROM: Premature preterm rupture of membranes
PVHI: Periventricular hemorrhagic infarction
ROP: Retinopathy of prematurity
SD: Standard deviation
