# Supplementary material for: Neonatal outcomes following previable rupture of membranes below 23 weeks’ gestation
Source: Eur J Pediatr. 2025 Jul 25;184(8):503. doi: 10.1007/s00431-025-06324-0 (PMC12296744; doi:10.1007/s00431-025-06324-0)
Supplement: Supplementary file 1 — Supplementary Material 1 (DOCX 17.1 KB) [file 431_2025_6324_MOESM1_ESM.docx]

**Supplemental Table A.**

Survival classified by gestational age at pPROM and gestational age at birth

| GA at pPROM | GA at birth | | | | | | | | |
| --- | --- | --- | --- | --- | --- | --- | --- | --- | --- |
|  | GA 22 ^0-7^ n=2 | GA 23 ^0-7^ n=29 | GA 24 ^0-7^  n=17 | GA 25 ^0-7^ n=21 | GA 26 ^0-7^ n=21 | GA 27 ^0-7^ n=12 | GA 28 ^0-7^ n=5 | GA 29 ^0-7^ n=2 | **total n=109** |
| GA 12 ^0-7^  n=1 |  |  |  | 1/1 (100%) |  |  |  |  | **1/1 (100.0%)** |
| GA 15 ^0-7^ n=2 |  |  |  |  | 2/2 (100%) |  |  |  | **2/2** **(100.0%)** |
| GA 16 ^0-7^  n=2 |  |  | 1/1 (100%) |  |  | 0/1 (0%) |  |  | **1/2** **(50.0%)** |
| GA 17 ^0-7^  n=4 |  | 0/2 (0%) |  | 0/1 (0%) |  | 1/1 (100%) |  |  | **1/4** **(25.0%)** |
| GA 18 ^0-7^  n=4 |  |  |  |  | 3/3 (100%) | 1/1 (100%) |  |  | **4/4** **(100.0%)** |
| GA 19 ^0-7^  n=2 |  |  | 0/1 (0%) |  | 1/1 (100%) |  |  |  | **1/2** **(50.0%)** |
| GA 20 ^0-7^  n=17 |  | 3/5 (60%) | 0/1 (0%) | 0/2 (0%) | 3/3 (100%) | 3/4 (75%) |  | 1/2 (50%) | **10/17 (58.8%)** |
| GA 21 ^0-7^  n=31 | 1/1 (100%) | 5/9 (56%) | 1/4 (25%) | 6/6 (100%) | 7/7 (100%) | 1/1 (100%) | 3/3 (100%) |  | **24/31 (77.4%)** |
| GA 22 ^0-7^  n=46 | 0/1 (0%) | 7/13 (54%) | 7/10 (70%) | 7/11 (64%) | 5/5 (100%) | 4/4 (100%) | 2/2 (100%) |  | **32/46 (69.6%)** |
| **total n=109** | **1/2** **(50.0%)** | **15/29 (51.7%)** | **9/17 (52.9%)** | **14/21 (66.7%)** | **21/21** **(100.0%)** | **10/12 (83.3%)** | **5/5** **(100.0%)** | **1/2** **(50.0%)** | **76/109 (69.7%)** |

**Abbreviations:** GA: gestational age (in weeks+days); pPROM preterm premature rupture of membranes

**Supplemental Table B.**

Survival without severe morbidity classified by gestational age at pPROM and gestational age at birth

| GA at pPROM | GA at birth | | | | | | | | |
| --- | --- | --- | --- | --- | --- | --- | --- | --- | --- |
|  | GA 22 ^0-7^ n=2 | GA 23 ^0-7^ n=29 | GA 24 ^0-7^  n=17 | GA 25 ^0-7^ n=21 | GA 26 ^0-7^ n=21 | GA 27 ^0-7^ n=12 | GA 28 ^0-7^ n=5 | GA 29 ^0-7^ n=2 | **total n=109** |
| GA 12 ^0-7^  n=1 |  |  |  | 1/1 (100%) |  |  |  |  | **1/1 (100.0%)** |
| GA 15 ^0-7^ n=2 |  |  |  |  | 2/2 (100%) |  |  |  | **2/2** **(100.0%)** |
| GA 16 ^0-7^  n=2 |  |  | 0/1 (0%) |  |  | 0/1 (0%) |  |  | **0/2** **(0.0%)** |
| GA 17 ^0-7^  n=4 |  | 0/2 (0%) |  | 0/1 (0%) |  | 1/1 (100%) |  |  | **1/4** **(25.0%)** |
| GA 18 ^0-7^  n=4 |  |  |  |  | 3/3 (100%) | 1/1 (100%) |  |  | **4/4** **(100.0%)** |
| GA 19 ^0-7^  n=2 |  |  | 0/1 (0%) |  | 1/1 (100%) |  |  |  | **1/2** **(50.0%)** |
| GA 20 ^0-7^  n=17 |  | 2/5 (40%) | 0/1 (0%) | 0/2 (0%) | 3/3 (100%) | 2/4 (50%) |  | 1/2 (50%) | **8/17 (47.1%)** |
| GA 21 ^0-7^  n=31 | 1/1 (100%) | 2/9 (22%) | 0/4 (0%) | 3/6 (50%) | 7/7 (100%) | 0/1 (0%) | 3/3 (100%) |  | **16/31 (51.6%)** |
| GA 22 ^0-7^  n=46 | 0/1 (0%) | 2/13 (15%) | 4/10 (40%) | 7/11 (64%) | 4/5 (80%) | 4/4 (100%) | 2/2 (100%) |  | **23/46 (50.0%)** |
| **total n=109** | **1/2** **(50.0%)** | **6/29 (20.7%)** | **4/17 (23.5%)** | **11/21 (52.4%)** | **20/21** **(95.2%)** | **8/12 (66.7%)** | **5/5** **(100.0%)** | **1/2** **(50.0%)** | **56/109 (51.4%)** |

**Abbreviations:** GA: gestational age (in weeks+days); pPROM preterm premature rupture of membranes. **Legend:** severe morbidity: IVH III, PVHI, cPVL, and/or ROP
